# In vitro screening of dihalomethanes as potential methane inhibitors in dairy cows

**DOI:** 10.3168/jdsc.2024-0700

**Published:** 2025-01-10

**Authors:** M. Thorsteinsson, M.O. Nielsen

**Affiliations:** Department of Animal and Veterinary Sciences, AU Viborg, Research Centre Foulum, Aarhus University, DK-8830 Tjele, Denmark

## Abstract

Volatile halogenated compounds are known to possess antimethanogenic properties with most in vivo studies focusing on trihalomethanes. However, concerns can be raised in relation to the use of these compounds as feed additives due to the transfer of halogenated metabolites into milk, which may pose a threat to consumer health. Hence, the objective of this in vitro study was to investigate the CH_4_-mitigating potential of 5 dihalomethanes (DHM) to potentially reduce the transfer of halogenated compounds when used as feed additives in the future. The DHM, dibromomethane (BM), bromoiodomethane (BIM), dichloromethane (CM), chloroiodomethane (CIM), and diiodomethane (DIIM) were dissolved in 99% ethanol, reaching final solutions of 2 m*M*. In 2 separate runs, 0.1 mL of the solutions were incubated as triplicates in buffered rumen fluid together with 0.5 ± 0.02 g (± SD) of corn silage. Similarly, corn silage and buffered rumen fluid with the addition of 0.1 mL of 99% ethanol served as control and blank samples, respectively. Accumulated gas production was determined using the ANKOM^RF^ system equipped with airtight gasbags. Gas composition in collected gas was determined after 24 h, whereas the concentration of VFA in the fermented rumen fluid was determined after 48 h of incubation. All DHM except CM resulted in significant reductions and increases in CH_4_ and H_2_ production, respectively, compared with the standard feed incubated without DHM. Hence, BIM, BM, and DIIM reduced CH_4_ production per gram of incubated DM by more than 90%, whereas CIM reduced CH_4_ production by 58% per gram of incubated DM. Reductions in CH_4_ production were accompanied by significant reductions in total VFA production, proportions of acetate in total VFA, and increases in proportions of propionate compared with the control treatment. In conclusion, BM, BIM, DIIM, and to a lesser extent CIM were efficient CH_4_ inhibitors; however, the DHM also had negative effects on fermentation parameters.

## Highlights

• Four out of five dihalomethanes reduced CH_4_ production from corn silage in vitro.
• Dibromomethane, bromoiodomethane, and diiodomethane reduced CH_4_ by more than 90%.
• Dichloromethane did not reduce CH_4_ production compared with the control treatment.
• Reductions in CH_4_ production were accompanied by reductions in total VFA production.

## Graphical Abstract

**Figure:**
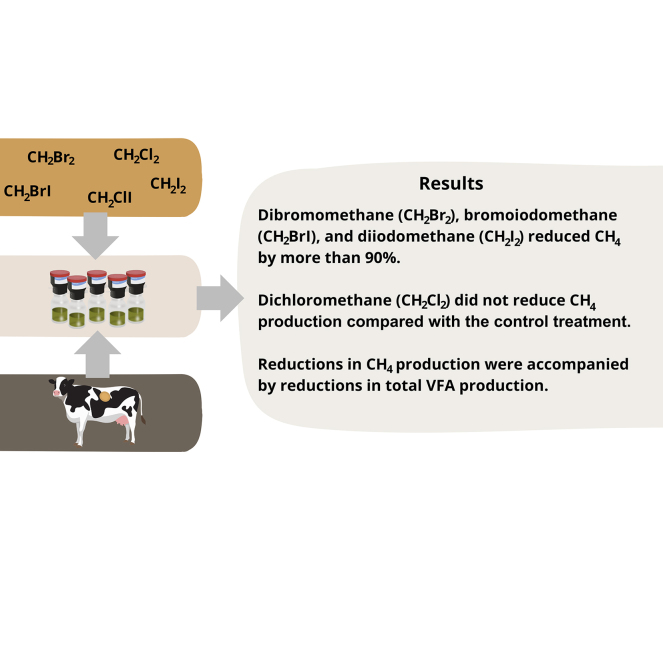
**Summary:** Halogenated compounds, particularly trihalomethanes, have demonstrated large potential as antimethanogenic feed additives. However, concerns can be raised in relation to the use of these compounds as feed additives due to the transfer of halogenated metabolites into milk. This in vitro study aimed to identify dihalomethanes with CH_4_-mitigating potential to potentially reduce the transfer of halogens to milk when used as feed additives. When added to corn silage, CH_4_ production was reduced by the dihalomethanes dibromomethane (>90%), bromoiodomethane (>90%), and chloroiodomethane (58%) compared with corn silage incubated alone in a buffered rumen inoculum, whereas dichloromethane showed no CH_4_-mitigating effect. However, reductions in CH_4_ production were accompanied by reductions in total volatile fatty acid (VFA) production; hence, future studies should focus on finding the optimal dose of the dihalomethanes resulting in the largest reduction without having negative effects on overall rumen fermentation.

Research investigating the effects of volatile halogenated compounds (**VHC**) as ruminal CH_4_ inhibitors has been conducted for several decades (Czerkawski and Breckenridge, 1975; Thorsteinsson et al., 2023a). The primary mode of action is believed to be the competitive binding of the compounds to enzymes in the final steps of methanogenesis (Wood et al., 1968), but VHC have also been suggested to inhibit CH_4_ production by serving as competing terminal electron acceptors (Yu and Smith, 2000). The antimethanogenic activity is usually related to the number of halogens on the molecule with iodine-containing compounds being the most efficient followed by brominated and chlorinated analogs (Chalupa, 1980). Hence, 30% to 66% reductions in enteric CH_4_ yield (g/kg DMI) have been reported when bromoform (extract from the red seaweed *Asparagopsis taxiformis*), chloroform, and iodoform were administered to dairy cows as antimethanogenic feed additives (Martinez-Fernandez et al., 2018; Thorsteinsson et al., 2023a; Alvarez-Hess et al., 2024). However, the use of VHC as antimethanogenic feed additives is not unproblematic as bromoform and chloroform can be categorized as potential human carcinogens (EPA, 2000; ECHA, 2024). Moreover, daily levels of supplemented iodine exceeded the maximum total limit set by the US Food and Drug Administration regulations (NRC, 2021) when iodoform was used as a CH_4_ inhibitor for dairy cows (Thorsteinsson et al., 2023a). Hence, concerns can be raised regarding the transfer of halogenated metabolites into milk, which may pose a threat toward consumer health (Krizsan et al., 2023). The aforementioned VHC are all trihalomethanes, indicating that the molecule consists of 3 halogens and 1 hydrogen covalently bound to 1 carbon atom. Transfer of halogens originating from halogenated CH_4_-mitigating feed additives into milk can, at least in theory, be reduced by reducing the number of halogen atoms on the molecule.

Therefore, the objective of the current study was to investigate the potential of 5 different dihalomethanes (**DHM**) as antimethanogenic feed additives in vitro. It was hypothesized that the DHM dibromomethane (CH_2_Br_2_; **BM**), bromoiodomethane (CH_2_BrI; **BIM**), dichloromethane (CH_2_Cl_2_; **CM**), chloroiodomethane (CH_2_ClI; **CIM**), and diiodomethane (CH_2_I_2_; **DIIM**) all would reduce CH_4_ production from a standard feed incubated in vitro in buffered rumen fluid, with the iodine-containing compounds being most efficient.

To compare the antimethanogenic potential of the 5 DHM (BM, BIM, CM, CIM, and DIIM), the compounds were evaluated on a molar basis. Hence, 2 m*M* solutions were prepared by dissolving the compounds in 99% ethanol. Due to the differences in molar masses of the compounds, this resulted in different wt/vol concentrations in the solutions (Table 1).

**Table 1:** Information on chemicals used in treatments

Item	Treatment^1^				
	BIM	BM	CIM	CM	DIIM
CAS no.	557–68–6^2^	74–95–3^2^	593–71–5^2^	75–09–2^2^	75–11–6^2^
Concentration (mg/mL)	0.44	0.35	0.35	0.17	0.54

1 BM = dibromomethane; BIM = bromoiodomethane; CM = dichloromethane; CIM = chloroiodomethane; DIIM = diiodomethane.

2 Purchased from Merck, Darmstadt, Germany.

In 2 separate runs, 0.1 mL of the solutions were added to Duran bottles (DWK Life Sciences, Mainz, Germany; capacity: 132.0 ± 1.1 mL) together with 0.5 ± 0.02 g of a standard feed, corn silage (ash: 3.54%; CP: 7.77%; NDF: 32.9%; starch: 35.1% on a DM basis). To quantify the CH_4_-mitigating potential of the treatments, corn silage (0.5 ± 0.02 g) with the addition of 0.1 mL of 99% ethanol was included as control samples, whereas buffered rumen fluid incubated alone with 0.1 mL of 99% ethanol served as blank samples. Each sample type was included as triplicates in both runs. To minimize possible confounding effects of bottle position and inoculation, the runs were divided into 3 blocks with one bottle per treatment in each block. The treatments were randomly assigned to a bottle within block.

Three rumen-cannulated nonlactating Danish Holstein cows (BW 755.6 ± 105.4 kg and BCS 3.75 ± 0.43) were used for collection of rumen fluid. The handling and care of the cows complied with the guidelines set out by the Danish Ministry of Environment and Food (Act No. 2028, 2020; Danish Ministry of Environment and Food, 2000) concerning animal experimentation and care of animals under experiments. The cows were fed 2 equal-sized meals of a diet consisting of grass-clover hay, barley straw, and a pelleted concentrate mixture (39.6% barley grain, 39.9% oat grain, 10% soybean meal, 3% rapeseed meal, 3% sugar beet molasses, and 4% of a commercial mineral mixture per kg fresh mixture) at maintenance level, which amounted to 7.7 kg of DM daily (with 13.7% starch, 13.9% CP, 24.1% NDF, and forage to concentrate ratio of 69:31 on a DM basis). Approximately 30 min before morning feeding, rumen liquid and particles were sampled and filtered through a nylon cloth with a pore size of 250 µm into preheated vacuum flasks. Buffer, redox indicator, reducing agent, and macro- and micromineral solutions were prepared and mixed as described by Menke and Steingass (1988). Rumen fluid was added in a 2:1 ratio (buffer solution: rumen fluid). After mixing, the solution was kept under anaerobic conditions by continuously flushing with N_2_. The final pH of buffered rumen fluid was 7.92 ± 0.10. Ninety milliliters of inoculum and 0.1 mL of additives were added to each bottle. Thereafter, a wireless fully automated ANKOM^RF^ module (ANKOM Technology, Macedon, NY) was mounted on each bottle for measurement of in vitro gas production, and the headspace was flushed with N_2_. The ANKOM^RF^ was operated as described by Thorsteinsson et al. (2023b). A gas-tight FlexFoil bag (1 L; SKC Ltd., Dorset, United Kingdom) was attached to each sensor module for the continuous collection of released gas. The bottles were incubated for 48 h at 38.5°C in a controlled incubator shaker (New Brunswick Excella E25R, Eppendorf, Hamburg, Germany) with the oscillation set at 50 rpm. After 24 h of incubation, 10 mL of gas was extracted from each gasbag using a gas-tight syringe with a twist valve (Hamilton Bonaduz AG, Bonaduz, Switzerland). Then the gas was transferred into Exetainer vials (Labco Limited, Ceredigion, United Kingdom) for later gas analyses. After 48 h of incubation, a sample of the fermented inoculum was collected for later VFA analysis by filtration of bottle contents through a F57 ANKOM bag (ANKOM Technology, Macedon, NY).

The corn silage was freeze-dried and ground on a 2-mm screen (Ultra Centrifugal Mill ZM 200, Verder Scientific, Hann, Germany). Nutrient content was analyzed using near-infrared spectroscopy at a commercial feed testing laboratory (Eurofins Agro Testing A/S, Vejen, Denmark). The collected inoculum was stabilized with 1 mL of 25% metaphosphoric acid (**MPA**) to reach 5% MPA in the stabilized sample. The concentrations of VFA were determined in stabilized inoculum after methanol-chloroform extraction with 2-ethylbutyrate as the internal standard, using GC (Trace 1310, Thermo Scientific, Germany) with a split/splitless injector at 225°C and a flame ionization detector at 250°C. A 30 m × 0.53 mm × 1 µm HP-FFAP column (Agilent Technologies Inc., Wilmington, DE) was used with helium as carrier gas at 34.5 kPa.

Concentrations of CH_4_ and H_2_ were determined in gas samples using GC (Trace 1310, Thermo Scientific, Germany) with a split/splitless injector at 150°C and a thermal conductivity detector at 200°C. A 30 m × 0.25 mm × 50 µm Rt-Q-BOND column (MicroQuartz, Munich, Germany) was used with argon as the carrier gas at 4 mL/min flow. The gas chromatograph was programmed with a gradually increased temperature starting at 80°C for 7 min and then from 80°C to 150°C at 20°C/min. Quantification was performed using standard curves with standard gases in concentrations ranging from 500 to 250,000 ppm for CH_4_ and from 250 to 50,000 ppm for H_2_.

Cumulative gas pressure was converted from pounds per square inch (psi) to milliliters (mL) of gas produced at standard temperature and pressure (IUPAC, 2014), assuming that the ideal gas law applies to the produced gases (predominantly CO_2_, CH_4_, and H_2_). From the cumulated total gas production (mL) and CH_4_ concentration (%) in collected gas after 24 h incubation, CH_4_ production (mL) was calculated. Total VFA production (mmol) after 48 h was calculated from concentration (mmol/L) and total inoculum volume in the bottles (L). Before the statistical analysis, total gas production parameters were blank-corrected.

The various response parameters were analyzed with the following linear mixed model in R 4.4.1 (R Core Team, 2024):

*Y_te_* = *µ* + *α_t_* + *A_e_* + *ε_te_*,

where *Y_te_* is the dependent response variable, *μ* is the overall mean, *α* is the fixed effect of treatment (*t* = 1–5), *A* is the random effect of experimental run (*e* = 1–2), and *ε_te_* is the random residual error, assumed to be independent with constant variance and normally distributed. Data are presented in tables as estimated marginal means (**EMS**) and SEM. Differences between EMS were evaluated using Tukey's method for comparison. Statistical significance was declared when *P* ≤ 0.05 and statistical tendency was declared when 0.05 < *P* ≤ 0.10.

Chalupa (1980) claimed that chlorinated VHC were less effective as CH_4_ inhibitors compared with iodinated and brominated VHC. This hypothesis seems to be supported by the results from the current study, at least when evaluated on a molar basis, as all DHM, except CM, significantly (*P* < 0.001) reduced CH_4_ per gram of incubated DM and OM compared with the control (Table 2). Moreover, BIM and DIIM resulted in significantly stronger suppression of CH_4_ production compared with CIM. These reductions were caused by a combination of both reduced total gas production and substantially lower CH_4_-percentage. However, it should be noted that wt/vol concentrations of the DHM in the solutions varied due to differences in molecular weights, causing the wt/vol concentration of DIIM to be 3 times as high as CM. In addition to the type of halogen on the molecules and concentration, the volatility of the compounds might also have affected the antimethanogenic potential of the DHM. The CM has a low boiling point (40°C), close to that of the incubation temperature, whereas the remaining compounds had boiling points ranging from 67 (DIIM) to 138°C (BIM; Merck, 2024). Hence, CM might have evaporated from the inoculum into the headspace relatively quickly after the initiation of the experiment and released from the bottles during the venting of the system. This hypothesis is supported by the similar CH_4_ between control and CM.

**Table 2:** Methane and hydrogen production after 24 h of incubation

Item	Treatment^1^						SEM	*P*-value
	BIM	BM	CIM	CM	DIIM	Control		
Total gas production after 24 h								
mL/g DM in corn silage	74.9^b^	70.8^b^	76.3^ab^	90.3^ab^	71.8^b^	98.1^a^	6.88	<0.01
mL/g OM in corn silage	77.1^b^	72.9^b^	78.6^ab^	93.0^ab^	73.9^b^	101.0^a^	7.09	<0.01
Methane after 24 h								
mL/g DM in corn silage	0.150^c^	0.929^bc^	4.63^b^	9.88^a^	0.463^c^	11.1^a^	0.973	<0.001
mL/g OM in corn silage	0.154^c^	0.957^bc^	4.76^b^	10.2^a^	0.446^c^	11.4^a^	1.00	<0.001
Concentration in total gas (%)	0.200^c^	1.34^c^	6.05^b^	10.9^a^	0.586^c^	11.1^a^	0.517	<0.001
Hydrogen after 24 h								
mL/g DM in corn silage	3.79^a^	2.92^a^	0.102^b^	0.025^b^	3.35^a^	0.022^b^	0.620	<0.001
mL/g OM in corn silage	3.90^a^	3.01^a^	0.105^b^	0.025^b^	3.45^a^	0.023^b^	0.638	<0.001
Concentration in total gas (%)	5.08^a^	4.68^a^	3.99^a^	0.127^b^	4.68^a^	0.022^b^	0.806	<0.001

a–c Values within the same row with different superscripts differ (*P* < 0.05).

1 BM = dibromomethane; BIM = bromoiodomethane; CM = dichloromethane; CIM = chloroiodomethane; DIIM = diiodomethane.

The H_2_ production followed the opposite pattern than CH_4_ with the highest production on BIM, BM, and DIIM. This change in gas composition was reflected in the VFA profile as the proportion of propionate (*P* < 0.001), formed in a [H]-consuming pathway, in total VFA increased, whereas the proportion of acetate (*P* < 0.001), formed in a net [H]-releasing pathway, in total VFA decreased causing the acetate:propionate ratio to decrease with increasing inhibition of CH_4_ production (*P* < 0.001; Table 3; Guyader et al., 2017). Valerate and caproate are normally considered to be [H]-sinks (Ungerfeld, 2015). Interestingly, while the proportion of propionate and butyrate appeared to follow the expected pattern toward a lower net [H]-release during reduced methanogenesis (Ungerfeld, 2020), the control treatment and CM had the highest proportion of caproate and valerate (*P* < 0.001 for both parameters). An increased ruminal H_2_ pressure has been speculated to change the ruminal thermodynamics and thereby negatively affect fermentation (Janssen, 2010; van Lingen et al., 2016). In the current study, the in vitro system was continuously vented during the incubation at a lower partial pressure than observed in the rumen (van Lingen et al., 2017). Hence, H_2_ accumulation cannot explain significantly lower total VFA production on all DHM treatments except CM. Therefore, it is possible that too high doses of DHM may have a negative impact on other ruminal microbes than the methanogenic archaea. A similar negative effect on ruminal digestibility was observed in vivo by Thorsteinsson et al. (2023a) in dairy cows administered increasing doses of iodoform intraruminally. Moreover, although microbial protein synthesis was not determined in the current in vitro study, the significantly (*P* < 0.001) lower proportion of the iso-fatty acid isovalerate could indicate a reduced efficiency of microbial protein synthesis.

**Table 3:** Volatile fatty acid production after 48 h of incubation

Item	Treatment^1^						SEM	*P*-value
	BIM	BM	CIM	CM	DIIM	Control		
Total VFA production after 48 h (mmol)	6.91^c^	7.24^b^	7.23^b^	7.74^a^	6.92^c^	7.97^a^	0.111	<0.001
% of total VFA								
Acetate	56.1^d^	58.9^c^	61.8^b^	68.0^a^	56.4^d^	68.3^a^	0.809	<0.001
Propionate	22.4^a^	20.6^b^	18.2^c^	12.9^d^	22.1^ab^	12.5^d^	0.430	<0.001
Butyrate	16.7^a^	15.8^ab^	14.6^b^	10.1^c^	16.7^a^	9.56^c^	0.477	<0.001
Valerate	1.55^b^	1.43^b^	1.47^b^	1.96^a^	1.54^b^	2.02^a^	0.0680	<0.001
Caproate	0.232^b^	0.266^b^	0.490^b^	3.35^a^	0.249^b^	3.67^a^	0.212	<0.001
Isobutyrate	1.06^a^	1.09^b^	1.09^b^	1.09^b^	1.10^b^	1.08^ab^	0.0243	<0.01
Isovalerate	1.91^b^	1.93^b^	2.30^ab^	2.64^a^	1.94^b^	2.80^a^	0.215	<0.001
Acetate:propionate ratio	2.51^d^	2.87^c^	3.41^b^	5.29^a^	2.56^cd^	5.45^a^	0.121	<0.001

a–d Values within the same row with different superscripts differ (*P* < 0.05).

1 BM = dibromomethane; BIM = bromoiodomethane; CM = dichloromethane; CIM = chloroiodomethane; DIIM = diiodomethane.

In conclusion, all DHM reduced CH_4_ production in vitro, except CM, with BIM, BM, and DIIM resulting in the largest reductions (>90%). The opposite pattern was observed for H_2_ production. Depression of CH_4_ production was associated with a negative effect on total VFA production. Hence, future studies should focus on finding the optimal dose of the DHM resulting in the largest CH_4_ reduction without negative effects on rumen fermentation.
